# Antineoplastic activity of rinvanil and phenylacetylrinvanil in leukaemia cell lines

**DOI:** 10.3892/ol.2014.1958

**Published:** 2014-03-10

**Authors:** AXEL LUVIANO, ITZEN AGUIÑIGA-SÁNCHEZ, PATRICIA DEMARE, REYNALDO TIBURCIO, EDGAR LEDESMA-MARTÍNEZ, EDELMIRO SANTIAGO-OSORIO, IGNACIO REGLA

**Affiliations:** 1 Laboratory of Drug Synthesis, L9-PA, Research Unit on Cell Differentiation and Cancer, L8-PB, Campus II, UMIEZ FES-Zaragoza, UNAM, Iztapalapa, Mexico City 09230, Mexico; 2 Hematopoiesis and Leukaemia Laboratory, Research Unit on Cell Differentiation and Cancer, L8-PB, Campus II, UMIEZ FES-Zaragoza, UNAM, Iztapalapa, Mexico City 09230, Mexico

**Keywords:** apoptosis, proliferative activity, cancer, vanilloid receptor, capsaicin

## Abstract

In the search for novel chemotherapeutic agents for cancer treatment, capsaicin has been shown to inhibit proliferation and induce apoptosis in various types of cancer cell line, including leukaemia cell lines. The capsaicin analogues, rinvanil and phenylacetylrinvanil (PhAR), share a binding affinity for vanilloid receptors and may have biological activities similar to capsaicin; however, their anticancer potential has not yet been reported. This study analyses the antineoplastic activities of rinvanil and PhAR in leukaemia versus normal cells. P388, J774 and WEHI-3 leukaemia cell lines, as well as mouse bone marrow mononuclear cells, were cultured with varying concentrations of rinvanil and PhAR. Following this, proliferation and apoptosis were determined by the sulforhodamine B (SRB) assay and DNA ladder. Cultured leukaemia cell lines and mouse bone marrow mononuclear cells demonstrated a dose-dependent inhibition of proliferation, while non-diseased cells were less sensitive to the cytotoxic effect of capsaicin, rinvanil and PhAR. Rinvanil and PhAR also induced apoptosis in leukaemia cell lines but not in bone marrow. Given the lower IC_50_ values for apoptosis induction in leukaemia cells compared with that of normal cells, PhAR is a promising selective anticancer agent.

## Introduction

A number of advances in the understanding of cancer on a biochemical, molecular, cellular and physiological level have been made in recent years and used in clinical diagnosis and the implementation of novel therapies. Despite this, there has been an increase in the number of new clinical cases of cancer, which is spurring the need for new medicines and treatment alternatives to tackle what is now a leading cause of mortality worldwide (1).

**Figure 1:**
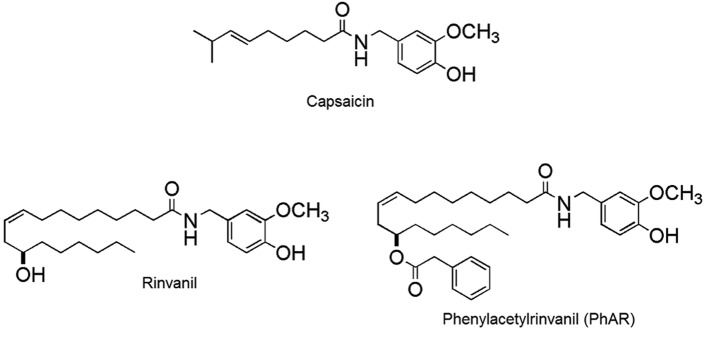
Chemical structures of capsaicin, rinvanil and phenylacetylrinvanil.

Capsaicin (N-[(4-hydroxy-3-methoxyphenyl)methyl]-8-methyl-(6E)-6-nonenamide; Fig. 1) is a naturally occurring organic compound and the main member of the capsaicinoid family, a group of compounds that give a characteristic pungency to the fruit of the chili pepper plant (*Capsicum spp.*). This effect is due to the agonist activity of the capsaicin receptor, transient receptor potential vanilla subfamily 1 (TRPV1). Expressed in sensory neurons, TRPV1 is a nonselective cation channel that is activated by heat (≥43°C) or protons, and also plays an important role in the transmission of pain impulses (2). Prolonged treatment with capsaicin desensitises neurons and thus generates an analgesic effect (3). Consequently, numerous studies have been investigating the role of capsaicin and its interaction with TRPV1 receptors in the treatment of a variety of pathologies that present with hyperalgesia, including rheumatoid arthritis (4,5), chronic neuropathic pain (6), diabetic neuropathy (7,8) and other neuralgias (9). In the search to identify new chemotherapeutic agents for cancer treatment, it has been revealed that capsaicin has inhibitory effects on cell proliferation and apoptosis induction in various types of cancer cell lines, including HepG2 (human hepatoma); AGS (gastric cancer); PC-3 (prostate cancer); MCF-7 (breast cancer); U373, U87, FC1 and FLS (glioma); NB4, UF-1, Kasumi-1, HL-60, K562, KU812 and U937 (leukaemia); and HT-29 cells (colon cancer) among others (10–16). Furthermore, several studies have demonstrated that capsaicin may have chemoprotective properties against certain carcinogenic and mutagenic agents (17,18) as well as the ability to induce terminal differentiation in A172 human glioblastoma cells (19). The evidence from these studies indicates the strong antitumour potential of capsaicin. The methods of obtaining capsaicin from natural sources are often inefficient and expensive, due to its low content in the fruits of the genus *Capsicum* and the presence of other compounds with similar polarity. In addition, due to its high pungency, it is difficult to manage its production by synthetic methods (20), which is an obstacle to its development as a potential chemotherapeutic agent. Two capsaicin analogues, rinvanil and phenylacetylrinvanil (PhAR) have been synthesised (Fig. 1). The latter is the most potent TRPV1 receptor agonist synthesised to date and is devoid of pungency (21,22); in fact, it is 1,000× more potent than capsaicin in its affinity for TRPV1 receptors (23).

While the structural and physiological associations between rinvanil, phenylacetylrinvanil (PhAR) and capsaicin are known, their antineoplastic effects have not yet been described. This study evaluates the antiproliferative and pro-apoptotic effects of rinvanil and PhAR on P388 mouse leukaemia cell lines, J774 and WEHI-3 leukaemia cell lines, and cultures of mononuclear cells from normal mouse bone marrow.

## Materials and methods

### Capsaicin and capsaicin analogues

Capsaicin was purchased from Sigma-Aldrich (St. Louis, MO, USA). Rinvanil and PhAR were synthesised and characterised as described in the study by Castillo *et al* (24).

### Animals

In this study, 12-week-old female BALB/c were used and maintained in pathogen-free conditions. Experiments were performed in the animal facility of FES-Zaragoza, National Autonomous University of Mexico (Iztapalapa, Mexico), in accordance with institutional guidelines. The mice were provided with autoclaved water and fed a standard powdered rodent diet *ad libitum*. All experimental protocols were approved by the ethics committee of FES-Zaragoza, National Autonomous University of Mexico in accordance with national regulations for the care and use of experimental animals.

### Cell culture

Total bone marrow cells of the mice were obtained from the femur by flushing with Iscove’s Modified Dulbecco’s Medium (IMDM) supplemented with 10% FBS. Mononuclear cells (MNCs) were obtained from the total cells via gradient separation with Ficoll-Paque (Amersham Biosciences AB, Uppsala, Sweden) at a density of 1.077 g/ml and washed twice with phosphate-buffered saline (PBS). MNCs were cultured for 120 h in IMDM (Gibco-BRL, Carlsbad, CA, USA) supplemented with 15% (v/v) FBS, 5% (v/v) horse serum (Gibco-BRL,) and 5 ng/ml recombinant mouse interleukin-3 (rmIL-3) (R&D System, Minneapolis, MN, USA). The cells were maintained in a humidified atmosphere containing 5% CO_2_ at 37°C and were maintained in a culture for a maximum of 120 h. P388, J774, and WEHI-3 mouse myeloid leukaemia cells were obtained from American Type Culture Collection (Manassas, VA, USA) and cultured in Iscove’s Modified Dulbecco’s Medium (Gibco-BRL) supplemented with 10% v/v foetal calf serum (Gibco-BRL) previously heat inactivated and kept at 37°C with 5% CO_2_ and saturating humidity.

### Cell proliferation assays

For proliferation assays, cells were grown in 96-well plates (Corning, Tewksbury, MA, USA) in the presence or absence of capsaicin, rinvanil and PhAR for 72 h under the culture conditions described above; proliferation was assessed with the sulforhodamine B (SRB) assay (Sigma-Aldrich) (25). Briefly, the cultured cells were fixed by adding 50 μl/well of cold trichloroacetic acid (TCA; Sigma-Aldrich) and incubated at 4°C for 1 h, followed by several washings to remove the TCA before the cell culture plate was allowed to dry at room temperature. The cells were then added to 100 μl/well of 0.4% SRB diluted in 1% acetic acid. After remaining under the stain for 20 min, several washes were performed with 1% acetic acid and the plate was allowed to dry at room temperature. The dye was solubilised with 50 μl/well of 10 mM Tris base (pH 10.4) and the plate was read on a spectrophotometer (Tecan Spectra A-5082; Tecan Austria GmbH, Grödig, Austria) at 570 nm.

### Cell viability

P388, J774 and WEHI-3 cell lines and normal mouse bone marrow cells were cultured in 96-well plates at cell densities of 3×10^4^, 5×10^3^, 1.3×10^4^ and 1×10^5^, respectively. They were then treated with or without IC_50_ concentrations of capsaicin, PhAR and rinvanil (Table I) and incubated for 72 h (leukaemia lines) or 120 h (mononuclear cells from normal mouse bone marrow). Finally, the cell suspensions were mixed with an equal volume of Trypan blue dye (Sigma-Aldrich) and counted directly under a light microscope (Axio Vert.A1, Primo Star Carl Zeiss, Göttingen, Germany). Unstained cells were considered as viable and stained cells nonviable (26).

### Apoptotic bodies

To assess the percentage of apoptotic bodies, the P388, J774 and WEHI-3 cell lines and mouse bone marrow cells, plated at a density of 1×10^5^ cells/ml, were treated with or without an IC_50_ concentration of capsaicin, rinvanil and PhAR. After 24 h of incubation, a sample of cells was fixed with methanol, stained with Giemsa dye and observed by light microscopy using a 100X objective.

### DNA fragmentation

To confirm the induction of apoptosis, DNA fragmentation was analysed. Briefly, P388, J774 and WEHI-3 cell lines (1×10^6^ cells/5 ml) were cultured in the presence or absence of IC_50_ concentrations of capsaicin, rinvanil or PhAR for 24 h. Cells were collected by centrifugation and washed twice with PBS. The cell pellet was resuspended in 0.3 ml lysis buffer [100 mM NaCl, 10 mM Tris-HCl (pH 8.0), 25 mM EDTA (pH 8.0), 0.5% SDS, 100 μg/ml proteinase K; Promega, Madison, WI, USA] and incubated while shaken at 37°C for 4 h. The lysate was treated with 100 μg/ml RNase (Promega) for 1 h at 37°C, followed by extraction with phenol-chloroform-isoamyl alcohol (25:24:1); the upper phase was collected and precipitated with absolute ethanol overnight at −20°C. DNA was collected by centrifugation (14,000 × g; 4°C; 20 min) and washed with 75% ethanol. The DNA pellet was resuspended in 100 μl TE buffer [1 mM EDTA (pH 8.0), 10 mM Tris-HCl (pH 8.0)] and incubated at 65°C for 1 h to facilitate solubilisation. Finally, the DNA in 0.5 mg/ml ethidium bromide was subjected to electrophoresis using a 2% agarose gel (Invitrogen Life Technologies, Carlsbad, CA, USA) at 60 V for 2.5 h and visualised under UV light (27).

The results are shown as the mean ± SD of three independent experiments with three replicates. Statistical significance was determined using one-way analysis of variance followed by Dunnett’s contrast. P<0.05 was considered to indicate a statistically significant difference.

## Results

### Rinvanil and PhAR inhibit the proliferation of leukaemia cell lines and mouse bone marrow cells

**Figure 2:**
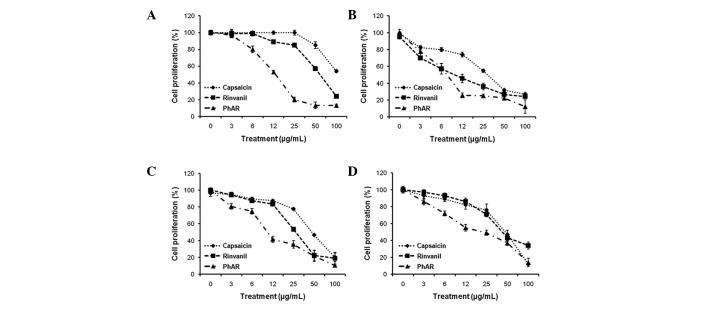
Antiproliferative effect of capsaicin, PhAR and rinvanil in (A) P388, (B) J774 and (C) WEHI-3 leukaemia cell lines and (D) mononuclear cells of normal mouse bone marrow. The cells were grown in 96-well plates with cell densities of 3×10^4^, 5×10^3^, 1.3×10^4^ and 1×10^5^ cells/ml, respectively, and treated with increasing concentrations of the compounds tested (0, 3, 6, 12, 25, 50 and 100 mg/ml). Cell proliferation was determined with the sulforhodamine B assay after 72 h incubation for leukaemia cell lines and 120 h for bone marrow cells. The results are shown as the mean ± SD of three independent experiments with three replicates each. PhAR, phenylacetylrinvanil.

P388, J774 and WEHI-3 leukaemia cell lines and normal mouse mononuclear bone marrow cells were cultured in 96-well plates at cell densities of 3×10^4^, 5×10^3^, 1.3×10^4^ and 1×10^5^, respectively, to demonstrate that rinvanil, PhAR and capsaicin (as a control), inhibit the proliferation of leukaemia cell lines, as well as mouse bone marrow cells, in a dose-dependent manner (Fig. 2). Based on the values of the average doses of inhibition (IC_50_), the leukaemia cell lines, except for P388 cells treated with capsaicin, were more sensitive than the normal bone marrow cells (Table I). Even the IC_50_ value of PhAR in P388 cells, 9.0 μg/ml, was increased to 40.7 μg/ml in bone marrow, providing evidence that leukaemia cells are 4.5-fold more sensitive compared with normal cells.

### PhAR is selectively cytotoxic to P388, J774 and WEHI-3 leukaemia cells

To determine if the decrease in proliferation was due to cell death induced by capsaicin, rinvanil and PhAR, cell viability was determined by evaluating membrane integrity with trypan blue dye exclusion. The P388, J774 and WEHI-3 leukaemia cells and normal mouse bone marrow cells were cultured with IC_50_ concentrations of capsaicin, PhAR and rinvanil, and incubated for 72 h (leukaemia lines) or 120 h (mononuclear mouse bone marrow). Our results show that in the case of J774 and P388 leukaemia lines, the three compounds significantly reduced cell viability with respect to the vehicle, although J774 cells were more sensitive, with viability percentages of only 55, 76 and 60% for cells treated with capsaicin, PhAR and rinvanil, respectively. In the case of WEHI-3 cells, capsaicin was the only agent to significantly reduce viability compared with cells treated with vehicle alone. Cells from normal mouse bone marrow had viabilities of 94 and 91% when treated with capsaicin and PhAR, respectively, without significant difference from vehicle, while rinvanil reduced viability to 80% (Table II). These results confirm that PhAR is selectively cytotoxic to leukaemia and similar to capsaicin (28,29).

### Rinvanil and PhAR induce cell death by apoptosis in P388, J774 and WEHI-3 cell lines, but not in mouse bone marrow cells

**Figure 3:**
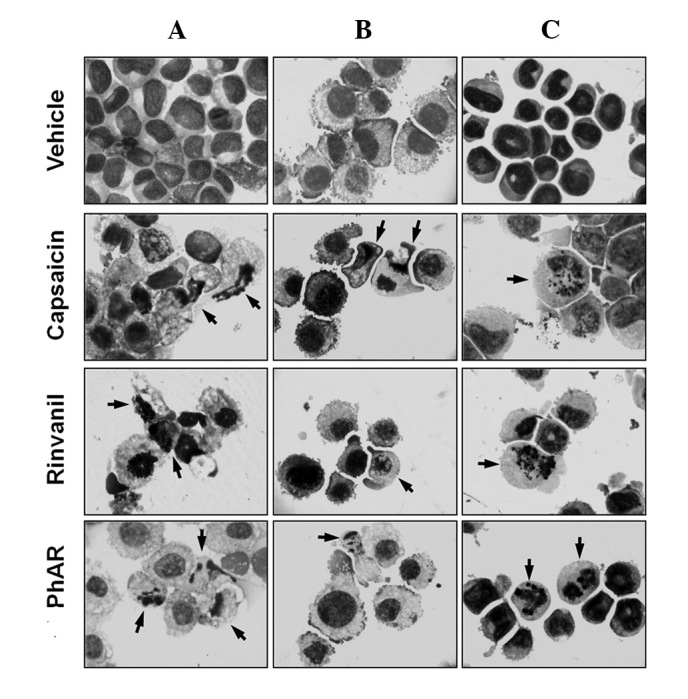
Morphology of apoptotic bodies of (A) J774, (B) P388 and (C) WEHI-3 cells. Leukaemia cell lines were cultured at a density of 1×10^5^ cells/ml and treated with or without IC_50_ concentrations of capsaicin, PhAR and rinvanil for 24 h. After treatment, cells were resuspended and applied to slides by cytospinning, fixed with methanol, stained with Giemsa and observed under light microscopy using a 100X objective. PhAR, phenylacetylrinvanil.

One important characteristic of cancer cells is their resistance to death by apoptosis (programmed cell death); apoptosis induction is an important parameter in determining the therapeutic antitumour potential of a compound (30). To assess whether PhAR and rinvanil induce cell death by apoptosis, P388, J774 and WEHI-3 cell lines and mouse bone marrow cells were cultured at a density of 1×10^5^ cells/ml with or without IC_50_ concentrations of PhAR and rinvanil, using capsaicin as a positive control for the induction of apoptosis. After 24 h of incubation for leukaemia cell lines or 120 h for cultured bone marrow cells, cells were stained with Giemsa and examined by light microscopy. The cell lines demonstrated characteristics of apoptosis (31), including an increase in cell volume, cytoplasmic modifications, change in nuclear size, nuclear chromatin condensation and nuclear fragmentation, as well as an intact cell membrane with the formation of apoptotic bodies (Fig. 3). Cultured mouse bone marrow mononuclear cells treated with these compounds showed no significant changes in cell morphology with respect to the vehicle. A quantification of the percentage of apoptotic bodies confirms that the leukaemia cell lines undergo apoptosis in response to PhAR and rinvanil treatment, while normal bone marrow cells do not (Table III).

As PhAR was demonstrated to have a lower IC_50_ for apoptosis induction, as observed by the formation of apoptotic bodies in cell lines, this molecule was used to confirm the induction of apoptosis by DNA fragmentation analysis.

**Figure 4:**
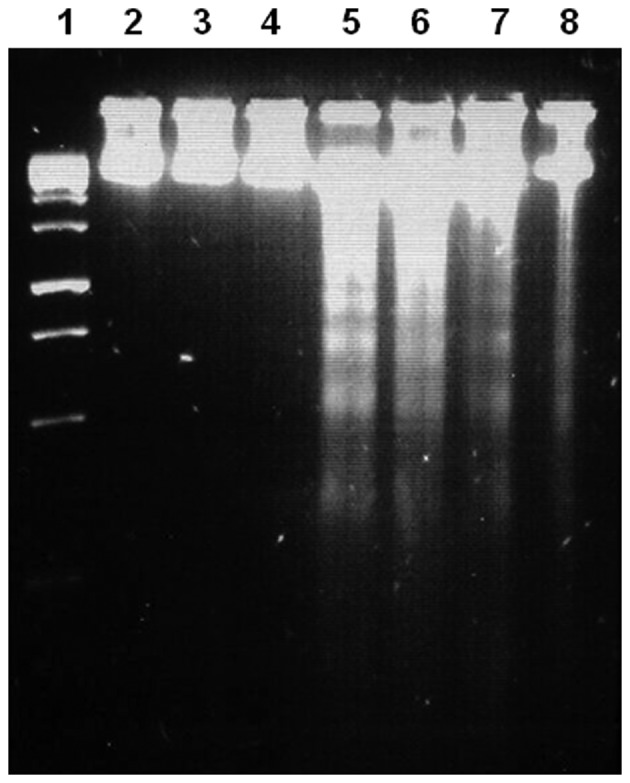
DNA fragmentation induced by PhAR in P388, J774 and WEHI-3 leukaemia cell lines, or by capsaicin in P388 cells. Lane 1, molecular weight marker; lanes 2–4, P388, J774 and WEHI-3 cells, respectively, treated with vehicle alone; lanes 5–7, P388, J774 and WEHI-3 cells, respectively, treated with IC_50_ concentrations of PhAR; lane 8, P388 cells treated with an IC_50_ concentration of capsaicin. P388, J774 and WEHI-3 cell lines (1×10^6^ cells/5 ml) were cultured for 24 h in the presence or absence of IC_50_ concentrations of PhAR, and P388 cells were treated with an IC_50_ concentration of capsaicin. At the end time of stimulation, DNA was extracted and visualised on an 2% agarose gel under UV light. PhAR, phenylacetylrinvanil.

For this, P388, J774 and WEHI-3 cells (1×10^6^ cells/5 ml) were cultured in the presence or absence of IC_50_ concentrations of PhAR for a period of 24 h; as a control, P388 cells were treated with an IC_50_ concentration of capsaicin. DNA was then extracted and visualised on an agarose gel under UV light (Fig. 4). These results show a typical pattern of internucleosomal DNA fragmentation of 180–200 bp in leukaemia cells treated with PhAR, as does capsaicin in P388 cells.

## Discussion

It is known that capsaicin can inhibit the proliferation of several human and mouse tumour cell lines without affecting normal cell proliferation, and that this antiproliferative effect is due to the induction of apoptosis (28,29). As rinvanil and PhAR are synthetic capsaicinoids, we evaluated the effect of these compounds on cell proliferation using capsaicin as a control. Based on the values of IC_50_, the leukaemia cell lines were more sensitive than the normal bone marrow cells (except for P388 treated with capsaicin). As the high potential for inhibition of proliferation observed with PhAR for normal and leukaemia cells correlates with its high affinity for vanilloid receptors, it would be of interest in the future to assess whether the difference in proliferative response is due to the density of receptors expressed in each cell type.

To determine whether the decrease in proliferation was due to cell death induced by capsaicin, rinvanil and PhAR, cell viability was determined by evaluating membrane integrity with trypan blue dye exclusion. The results confirm that PhAR is selectively cytotoxic to leukaemia, similar to capsaicin (28,29).

Capsaicin, the pungent component present in chili pepper, has potential anti-inflammatory, antioxidant, antiproliferative and anticancer properties; it also has chemopreventive effects against chronic inflammatory diseases, including cancer (31). Rinvanil and PhAR are synthetic capsaicinoids that have powerful cytotoxic and apoptotic effects on leukaemia cells and as they lack pungency, they may act as improved antineoplastic agents over the naturally occurring capsaicin compound. PhAR, in particular, stands out for its greater cytotoxic activity, which inhibits the proliferation of leukaemia cells by promoting apoptosis. The majority of drugs currently used for cancer chemotherapy, produce side effects such as immunosuppression and anaemia which are in many cases permanent or irreversible and lethal (33,34), at this time the effect of PhaR *in vivo* is unknown, in the future these events will be analyzed.

In conclusion, PhAR and rinvanil inhibit proliferation of leukaemia lines and bone marrow mononuclear cells in a dose-dependent manner, although the latter were less sensitive to the cytotoxic effect of rinvanil and PhAR, as well as capsaicin. Inhibition of cell proliferation was due to the induction of cell death by apoptosis in leukaemia lines but not in normal cells. The characteristics of low pungency and selective toxicity toward leukaemia cells suggests that both rinvanil and particularly PhAR may be biomedically relevant and promising anticancer agents.

## Figures and Tables

**Table I:** IC_50_ values for the different leukaemia cell lines and mononuclear cells of normal mouse bone marrow.

	IC_50_ (μg/ml)^a^			
	
Treatment	P388	J774	WEHI-3	Bone marrow
Capsaicin	72.1±1.8	32.5±1.7	47.7±2.5	53.5±3.1
Rinvanil	49.2±2.2	10.2±3.0	31.2±3.6	72.6±2.4
PhAR	9.0±2.0	8.0±3.7	3.0±3.1	40.7±2.4

a Necessary concentration to inhibit cell proliferation by 50%.

PhAR, phenylacetylrinvanil.

**Table II:** Cell viability (%) of the leukaemia cell lines and mononuclear cells of normal mouse bone marrow treated with the IC_50_ concentrations of capsaicin, rinvanil and PhAR.

	Cell viability (%)		
	
Treatment	Capsaicin	Rinvanil	PhAR
(A)			
Vehicle	97±2.8	97±2.8	97±2.8
IC_50_	80±9.0^a^	93±6.2^a^	93±3.8^a^
(B)			
Vehicle	98±1.9	98±1.9	98±1.9
IC_50_	55±6.2^a^	76±6.9^a^	60±8.0^a^
(C)			
Vehicle	98±4.2	98±4.2	98±4.2
IC_50_	86±5.0^a^	91±7.4	91±7.7
(D)			
Vehicle	97±3.3	99±3.3	99±3.3
IC_50_	94±2.2	80±6.3^a^	91±3.7

Percent viability of (A) P388, (B) J774, (C) WEHI-3 leukaemia cell lines and (D) bone marrow mononuclear cells of normal mice treated with IC_50_ concentrations of capsaicin, PhAR and rinvanil. Cell cultures were stimulated at time zero and after 3 days of incubation the viability was assessed by trypan blue exclusion. The results are shown as the mean ± SD for three independent experiments with six replicates.

a P<0.05 compared with vehicle (0 mg/ml), according to analysis of variance with Dunnett’s test.

PhAR, phenylacetylrinvanil.

**Table III:** Percentage of apoptotic bodies in different leukaemia cell lines and mononuclear cells of normal mouse bone marrow.

	Apoptotic bodies (%)			
	
Treatment	P388	J774	WEHI-3	Bone marrow
Vehicle	0	2±1	0	5±3
Capsaicin	41±2^a^	31±3^a^	17±3^a^	6±4
Rinvanil	41±3^a^	30±3^a^	15±2^a^	7±2
PhAR	42±5^a^	37±2^a^	16±2^a^	6±4

The results are shown as the mean ± SD of three independent experiments with three replicates.

a P<0.05 compared with vehicle (0 mg/ml), according to analysis of variance with Dunnett’s test.

PhAR, phenylacetylrinvanil.

## References

[1] World Health Organization (2010). World health statistics.

[2] Caterina MJ, Schumacher MA, Tominaga M, Rosen TA, Levine JD, Julius D (1997). The capsaicin receptor: a heat-activated ion channel in the pain pathway. Nature.

[3] Winter J, Bevan S, Campbell EA (1995). Capsaicin and pain mechanisms. Br J Anaesth.

[4] McCarthy GM, McCarty DJ (1992). Effect of topical capsaicin in the therapy of painful osteoarthritis of the hands. J Rheumatol.

[5] Matucci-Cerinic M, McCarthy G, Lombardi A, Pignone A, Partsch G (1995). Neurogenic influences in arthritis. J Rheumatol.

[6] Sindrup SH, Jensen TS (1999). Efficacy of pharmacological treatments of neuropathic pain: an update and effect related to mechanism of drug action. Pain.

[7] Low PA, Opfer-Gehrking TL, Dyck PJ, Litchy WJ, O’Brien PC (1995). Double-blind, placebo-controlled study of the application of capsaicin cream in chronic distal painful polyneuropathy. Pain.

[8] Ross DR, Varipapa RJ (1989). Treatment of painful diabetic neuropathy with topical capsaicin. N Engl J Med.

[9] Szallasi A, Blumberg PM (1999). Vanilloid (Capsaicin) receptors and mechanisms. Pharmacol Rev.

[10] Huang SP, Chen JC, Wu CC (2009). Capsaicin-induced apoptosis in human hepatoma HepG2 cells. Anticancer Res.

[11] Chow J, Norng M, Zhang J, Chai J (2007). TRPV6 mediates capsaicin-induced apoptosis in gastric cancer cells-mechanisms behind a possible new ‘hot’ cancer treatment. Biochim Biophys Acta.

[12] Sánchez AM, Malagarie-Cazenave S, Olea N, Vara D, Chiloeches A, Díaz-Laviada I (2007). Apoptosis induced by capsaicin in prostate PC-3 cells involves ceramide accumulation, neutral sphingomyelinase, and JNK activation. Apoptosis.

[13] Chou CC, Wu YC, Wang YF, Chou MJ, Kuo SJ, Chen DR (2009). Capsaicin-induced apoptosis in human breast cancer MCF-7 cells through caspase independent pathway. Oncol Rep.

[14] Amantini C, Mosca M, Nabissi M (2007). Capsaicin-induced apoptosis of glioma cells is mediated by TRPV1 vanilloid receptor and requires p38 MAPK activation. J Neurochem.

[15] Ito K, Nakazato T, Yamato K (2004). Induction of apoptosis in leukemic cells by homovainillic acid derívate, capsaicin, through oxidative stress: implication of phosphorylation of p53 at Ser-15 residue by reactive oxygen species. Cancer Res.

[16] Kim YM, Hwang JT, Kwak DW, Lee YK, Park OJ (2007). Involvement of AMPK signaling cascade in capsaicin-induced apoptosis of HT-29 colon cancer cells. Ann NY Acad Sci.

[17] Surh YJ, Lee E, Lee JM (1998). Chemoprotective properties of some pungent ingredients present in red pepper and ginger. Mutat Res.

[18] Surh YJ, Lee RC, Park KK, Mayne ST, Liem A, Miller JA (1995). Chemoprotective effects of capsaicin and diallyl sulfide against mutagenesis or tumorigenesis by vinyl carbamate and N-nitrosodimethylamine. Carcinogenesis.

[19] Gil YG, Kang MK (2008). Capsaicin induces apoptosis and terminal differentiation in human glioma A172 cells. Life Sci.

[20] Appendino G, Fattorusso E, Taglialatela-Scafati O (2008). Capsaicin and capsaicinoids. Modern Alkaloids: Structure, Isolation, Synthesis and Biology.

[21] Appendino G, De Petrocellis L, Trevisani M (2005). Development of the first ultra-potent “capsaicinoid” agonist at transient receptor potential vanilloid type 1 (TRPV1) channels and its therapeutic potential. J Pharmacol Exp Ther.

[22] Appendino G, Minassi A, Morello AS, De Petrocellis L, Di Marzo V (2002). N-Acylvanillamides: development of an expeditious synthesis and discovery of new acyl templates for powerful activation of the vanilloid receptor. J Med Chem.

[23] Voets T, Droogmans G, Wissenbach U, Janssens A, Flockerzi V, Nilius B (2004). The principle of temperature-dependent gating in cold- and heat-sensitive TRP channels. Nature.

[24] Castillo E, Regla I, Demare P, Luviano-Jardón A, López-Munguía A (2008). Efficient chemoenzymatic synthesis of phenylacetylrinvanil: an ultrapotent capsaicinoid. Synlett.

[25] Vichai V, Kirtikara K (2006). Sulforhodamine B colorimetric assay for cytotoxicity screening. Nat Protoc.

[26] Strober W (2001). Trypan blue exclusion test of cell viability. Curr Protoc Immunol (Appendix 3).

[27] Gross-Bellard M, Oudet P, Chambon P (1973). Isolation of high-molecular-weight DNA from mammalian cells. Eur J Biochem.

[28] Morré DJ, Chueh PJ, Morré DM (1995). Capsaicin inhibits preferentially the NADH oxidase and growth of transformed cells in culture. Proc Natl Acad Sci USA.

[29] Morré DJ, Sun E, Geilen C (1996). Capsaicin inhibits plasma membrane NADH oxidase and growth of human and mouse melanoma lines. Eur J Cancer.

[30] Call JA, Eckhardt SG, Camidge DR (2008). Targeted manipulation of apoptosis in cancer treatment. Lancet Oncol.

[31] Fenech M (2007). Cytokinesis-block micronucleus cytome assay. Nat Protoc.

[32] Oyagbemi AA, Saba AB, Azeez OI (2010). Capsaicin: a novel chemopreventive molecule and its underlying molecular mechanisms of action. Indian J Cancer.

[33] Griffin AM, Butow PN, Coates AS (1996). On the receiving end V: Patient perceptions of the side effects of cancer chemotherapy in 1993. Ann Oncol.

[34] Carey MP, Burish TG (1988). Etiology and treatment of the psychological side effects associated with cancer chemotherapy: a critical review and discussion. Psychol Bull.

